# Supplementary material for: Patterns of Intron Gain and Loss in Fungi
Source: PLoS Biol. 2004 Nov 30;2(12):e422. doi: 10.1371/journal.pbio.0020422 (PMC532390; doi:10.1371/journal.pbio.0020422)
Supplement: Table S1 — Also available at http://genes.mit.edu/NielsenEtAl/. (4.3 MB ZIP). [file pbio.0020422.st001.zip › NielsenEtAl/html/373.html]

AN2063.1.NCU03981.1.MG02504.1.FG09466.1


```
 CLUSTAL W (1.82) Multiple Sequence Alignments - Introns Inserted


Sequence 1: NCU03981.1	779 aa
Sequence 2: MG02504.1	791 aa
Sequence 3: FG09466.1	776 aa
Sequence 4: AN2063.1	810 aa
Alignment Length: 827 aa
Number Identitical Residues: 267 aa
Alignment Score (without introns) 13810


MG02504.1 	MSRHSAFNNYDYDNELDEYDG--YDNTGEEELSPEDREQMVLGTSAVREALAAESSKVTT
NCU03981.1	MARHQNIRNLDYEAELEEYGA--FSDEEEEELSPEDQVRMREGTAQVLEALGVEAHKVPK
FG09466.1 	MSRHRIVHTFDTNDIVSEFDGDDYEEEGEDELSPEDRQAMDQGTAEVRAALGTEANKVTT
AN2063.1  	MSRHRVKAVSYDDDDYDDYDD------GYDSQDPEEREFLEECTREVQNQLAAGDPPVSA
          	*:**        :   .::.         :. .**::  :   *  *   *..    *. 

MG02504.1 	A--QIQEALWHYYYDVDKSVAYLVS~KYISPPADSKKAKKAT~NNPE~GM2DSAWSGGYG
NCU03981.1	T--QIEESLWHYYWDVDKTITYLIS~KYIDPPKKPAKTAPPK~TAPK~--~---------
FG09466.1 	T--QIEEALWHYYYDVDKSVTYLIK~TFIAPAPKPAKKTPED1HGRL~SS~G--------
AN2063.1  	TRDEVQDALWHYYNDVDKSVNYLRG1KKTKEADKKAAAAKAK~VNAV1WA~RVCKPVSTG
          	: .:::::***** ****:: **   .    . .                   . . . .

MG02504.1 	AV~PEA~AKASAFRP-----PMSFWFDDMPWLNIPDHRRA-EFIAPELPRGGLLGGS--G
NCU03981.1	--~QDA1AIASASRQ-----SFAHLFRDLPWGNIPPERQA-VLIPPPQLPAGLLGGS--G
FG09466.1 	-Y~NFN~MKLPPVDK-----VPACFFNDMPWLNIPQERQT-HFIEPERPRGGLLGGG--E
AN2063.1  	EM1PAY~PIPPTTATTHPHFSAADFFRDSPWLNIPAHRKADILVEPLYPRLGLLGGAPES
          	          ..   : .    :  * * ** *** .*::. :: *     *****... 

MG02504.1 	AAPKMSKLQALAAARKKKASDKK-----FEETEPSPEQQMGSLSLNS-----SEIKLPAT
NCU03981.1	APPKMSKLQQLAAARKKKAEEKS-----ASGEVEQTQVKMTELTVND-----NQASKENR
FG09466.1 	GAPKMSKLQALAAARKKKTEEK--------KEQEKLEKGVSKLSVAG-----SEKENQSP
AN2063.1  	GGGKMSKLAALAAARKKKEGDRSPASTTGTDAQSPSEAKGATLSLRERLAASKQVKQESL
          	.  *****  ********  ::..::::        :     *::    :::.: .    

MG02504.1 	PKLGALAKRRNEAASVSSDYRPPQSVVMSQPEVPTMDGAADEMGTRTTSNETELNTNVV-
NCU03981.1	PLAGVFGKRVKISETTAQGRIPLTMAEPTRPEIPQAQAPEATPSQHQEEDPIDPEPAVAP
FG09466.1 	-----FNQRQQIPTPASQAQPTQYDMDIDHEENEYKKPTPAHKDEFTLDSFPGVEIVTG-
AN2063.1  	PSLQSLKRRSEQSQPPKKSPEPKPSVPAATGLEDSIKSVQIEDNRHQEEKEQHVNIRAP-
          	.    : :* : . .  .   .              .      .    ..    :  .  

MG02504.1 	--AEPSAFACTLLGPRS---ISVSSHKQQQQSFGLPYLVMDPSISDIFSRPSPDDRVMTA
NCU03981.1	PKAQPSAFASTLFGPS-----SDSPKRRPREVFALPYMAISSYSPDAFGKPSPDDVVLAA
FG09466.1 	--PNPSALSRTLFGSA-------PANTQKPDVFAMPYTSSPSFLAQAFAEPSPDDIVFAA
AN2063.1  	----PSTFAATIVGDDIRPKMTEPSHLYTNTLDLIRIYGQDLAEPFDFSGPSPDDVILNA
          	    **::: *:.*    .. : ..:        :         .  *. ***** :: *

MG02504.1 	QAQA1GK~----PKATAKKGK-SDKVDSLSNSVQSLKVEDVAPLPKSKNLDVLGEYEKSI
NCU03981.1	QAKA1GK~----KSANVHNKKGSSSADGVTNQVSQLKIDDT-PLPKSRNLNVLSEFEKQK
FG09466.1 	QAKA1GK~QPAPKAAKKPQKEKVKDASEAEKDVAGLKIVDA-PPPKSKGLDVLKEYENSS
AN2063.1  	QSSA~KG1--FKSKQPAPKPQGDRKAAGLAGDMGNLSVAEK-VNVKSKNLDVLSEYKKSK
          	*:.*              : :   ..     .:  *.: :     **:.*:** *:::. 

MG02504.1 	KKKNASFVVVG~HVDAGKSTMMGRLLLDMNVVDDRTISKYKKEAEAMGKGSFALAWVLDS
NCU03981.1	GKKTASFVVVG~HVDAGKSTMMGRLLLDLNVVDQRTVDKLRKEAEKIGKTSFALAWVLDQ
FG09466.1 	NKRSISFVVVG1HVDAGKSTLMGRLLLELKFVEKHTIDRYRKQAEKSGKQSFALAWVMDQ
AN2063.1  	RKNAMNFVVIG1HVDAGKSTLMGRLLADLKAVDQRTLDKYRREAEKIGKGSFALAWVLDQ
          	 *.  .***:* ********:***** ::: *:.:*:.: :::**  ** *******:*.

MG02504.1 	TSDERAHGVTIDIAKSRFETESTIFTILDAPGHQDFVPNMIAGASQADFAILVIDATVGA
NCU03981.1	RHEERSRGVTIDIATNRFETETTSFTILDAPGHRDFIPNMIAGASQADFAILVIDASTGA
FG09466.1 	RSEERERGVTIDIATNHFETEKTSFTILDAPGHRDFVPNMIAGASQADFAILVIDANTGA
AN2063.1  	GSEERARGVTIDIATRKFETENTVFTIVDSPGHRDFVPNMIAGASQADFAVLVIDASIGN
          	  :** :*******. :****.* ***:*:***:**:*************:*****. * 

MG02504.1 	YERGLKGQTKEHAQLIRSIGVSRIIVAVNKLDATNWSQDRFNEISDGMSGFMSALGFQMK
NCU03981.1	FESGLKGQTREHSLLIRSMGVSRIIVAVNKLDTVNWSQERFDEITHQVSGFLTATGFQPK
FG09466.1 	YEKGLKGQTREHVLLLRSLGVQRLVIAVNKLDMVGWSQERYDEIAQQVSGFLAGLGFVSK
AN2063.1  	YESGLKGQTKEHALLVRSMGVQRIIVAVNKMDTVQWDFGRFEEIEQQVSAFLITAGFQAR
          	:* ******:**  *:**:**.*:::****:* . *.  *::** . :*.*:   **  :

MG02504.1 	NISFIPLSGLNGDNMVKRSTAEAASWYTGPTLLEELENSEPM-TRALKEPLRITVSDIYN
NCU03981.1	NIAFVPVSGLHGDNLVRKSTDPAASWYTGKTLVEELEASEPS-ARALAKPLRMTISEVMR
FG09466.1 	NIDFVPISGLNGDNLARRTEDPAASWYTGPTLIEALENSEPTTARALKSPFRMAISEVFR
AN2063.1  	NICFVPCSGVNGDNITRRSEDPNVSWYNGRTLVEELEETKPY-FHALDKPLRMTIGDVFR
          	** *:* **::***:.:::    .***.* **:* ** ::*   :** .*:*:::.:: .

MG02504.1 	-IGQSTLTVGGRLDAGSVQMGDALLVQPSGEKAYIKTIEVDSND-VDWAVAGQNVLLQLS
NCU03981.1	-TPQSSISITGRIDAGSLQMGDALLVQPSGEKAYVKSLQVDDGEPADWAVAGQNVVLHLS
FG09466.1 	-SQLGTTTIAGRVDAGSFQIGDALLVQPSGEEAYVKSIMVDSDM-QDWAVAGQNVSVALT
AN2063.1  	GSIQNPLSISGRIDAGSLQVGDQILTMPSGETATIRSLEVDEEP-NDWAVAGQNVVLHLA
          	.   .. :: **:****.*:** :*. **** * :::: **.    ********* : *:

MG02504.1 	HIDPERVRAGDVICDPTNPIQCIDSFTAKVLAFDHLWPMPLQVHRGRLDAVGTLQSMLAV
NCU03981.1	NIDPIHVRVGDIVCDPAKPIQCVDTFTLKALAFDILMPMQVDVHRGRLHAAGKIEAIDAI
FG09466.1 	NIDPIHIRVGDMLCPTKNPINCSDNFVMKAMAFEHLMPMPVDLHRGRLHSAGQIVSIAAT
AN2063.1  	NIDPIHLRSGDVVCHASAPIPNITSFTCKVLAFDHLLPSMVDIHRGRLHVPGRISKLVAT
          	:*** ::* **::* .  **    .*. *.:**: * *  :::*****.  * :  : * 

MG02504.1 	LDNATGTVVKKKPKIVKPGMTARVVIKMSGTKVPLEAGQRIILRCDGRTLAAGIIE
NCU03981.1	LDKVTGKVTKKKPMIVKPGTVSRVRVTLH-SKVPLEAGQRVVLRSGGQTVAAGLLE
FG09466.1 	LDKVTGAVVKKKARVVQPGGVARVSVKLA-AKVPLEAGQRVVIRSGGETVAAGLLE
AN2063.1  	LDKGSGNVLKKKPKIVQPGAVARIVVELD-QAVPLEAPTRIILRAGGDTVAAGLLE
          	**: :* * ***. :*:** .:*: : :    *****  *:::*..* *:***::*
```
